# Inoculation with *Micromonospora* sp. enhances carbohydrate and amino acid production, strengthening antioxidant metabolism to mitigate heat stress in wheat cultivars

**DOI:** 10.3389/fpls.2024.1500894

**Published:** 2024-12-19

**Authors:** Abdelrahim H. A. Hassan, Enas Shaban Ahmed, Mohamed S. Sheteiwy, Yousef Alhaj Hamoud, Mohammad K. Okla, Amal Mohamed AlGarawi, Maria Gabriela Maridueña-Zavala, Ibrahim A. Alaraidh, Ahmed M. Reyad, Hamada Abdelgawad

**Affiliations:** ^1^ School of Biotechnology, Nile University, Giza, Egypt; ^2^ Department of Food Safety and Technology, Faculty of Veterinary Medicine, Beni-Suef University, Beni-Suef, Egypt; ^3^ Botany and Microbiology Department, Faculty of Science, Beni-Suef University, Beni-Suef, Egypt; ^4^ Department of Integrative Agriculture, College of Agriculture and Veterinary Medicine, United Arab Emirates University, Abu Dhabi, United Arab Emirates; ^5^ Department of Agronomy, Faculty of Agriculture, Mansoura University, Mansoura, Egypt; ^6^ The National Key Laboratory of Water Disaster Prevention, College of Hydrology and Water Resources, Hohai University, Nanjing, China; ^7^ Botany and Microbiology Department, College of Science, King Saud University, Riyadh, Saudi Arabia; ^8^ Centro de Investigaciones Biotecnológicas del Ecuador (CIBE), Escuela Superior Politécnica del Litoral, ESPOL, Guayaquil, Ecuador

**Keywords:** wheat cultivars, heat stress, sugars, anthocyanin metabolism, phenylpropanoid pathways, ASC/GSH cycle 2

## Abstract

**Introduction:**

Heat stress caused by global warming adversely affects wheat yield through declining most nutritional quality attributes in grains, excluding grain protein content.

**Methods:**

This research investigated the biochemical, physiological, and antioxidant responses of wheat plants under heat stress, focusing on the role of plant growth-promoting bacteria (*Micromonospora* sp.). Two wheat genotypes were studied: one heat-sensitive and one heat-tolerant, examining their responses to heat stress with and without bacterial inoculation.

**Results:**

Under heat stress, the sensitive cultivar experienced significant reductions in photosynthesis rate, chlorophyll content, and RuBisCO activity (57-61%), while the tolerant cultivar had milder reductions (24-28%). *Micromonospora* sp. treatment notably improved these parameters in the sensitive cultivar (+48-78%), resulting in a substantial increase in biomass production (+43-53%), which was not seen in the tolerant cultivar. Additionally, oxidative stress markers (H_2_O_2_ and MDA) were elevated more in the sensitive cultivar (82% and 90% higher) compared to the tolerant one. *Micromonospora* sp. treatment effectively reduced these markers in the sensitive cultivar (-28% and -27%). Enhanced activity of antioxidant enzymes and ASC-GSH pathway enzymes was particularly evident in *Micromonospora* sp.-treated sensitive plants. Carbohydrate metabolism shifted, with increased soluble sugars and significant rises in sucrose content in *Micromonospora* sp.-treated plants under stress.

**Discussion:**

The higher soluble sugar levels facilitated amino acid synthesis, contributing to biosynthesis of secondary metabolites, including flavonoids, polyphenols, and anthocyanins. This was reflected in increased activity of phenylalanine ammonia-lyase, cinnamate (CA) 4-hydroxylase, and chalcone synthase enzymes, indicating the activation of phenylpropanoid pathways. Overall, the findings suggest that *Micromonospora* sp. can mitigate heat stress effects by enhancing photosynthetic efficiency, antioxidant defense, and metabolic adaptations in heat-sensitive wheat cultivars.

## 1 Introduction

According to Intergovernmental Panel for Climate Change (IPCC), earth’s surface temperature from 2011-2020 increased by 1.1°C compared to the period of 1850-1900, primarily due to human activities through emissions of greenhouse gases (IPCC, 2023). Based on the present acceleration, the earth’s temperature is expected to increase by an additional 1.5°C by 2050 (IPCC, 2023). Over the past decade, most research in the agronomy section has emphasized understanding the effect of such deviations in normal temperature on plant growth and production, which can cause stress such as heat, salt, drought and water deficit (Gui et al., 2021; Sheteiwy et al., 2021). Previous research has indicated that heat stress caused by global warming adversely affected wheat yield through declining most nutritional quality attributes in grains, excluding grain protein content, which can improve under higher temperatures in some plants (Zhao et al., 2022; Kumar et al., 2023). In this regard, a reduction of about 6% in wheat grain yield is expected for every degree Celsius of temperature rise, based on a crop simulation modeling analysis (Asseng et al., 2015). Similarly, it has previously been reported that there is an unambiguous relationship between wheat production and air temperature during the growth season, so for the temperature rises by 1.5°C and 3°C, a predicted decline in wheat yields is expected at about 7% and 24%, respectively (Hussain and Mudasser, 2007). Moreover, changes in the morphologic and physiologic parameters of crops were reported when they were exposed to even short period of heat stress (Ullah et al., 2022). The production of free radicals in plant cells has been previously reported as a result of macromolecules oxidation (Notununu et al., 2022). Thus, detriment to photosynthetic systems and a decline in photosynthesis rate can occur in response to free radicals and their negative impacts on cell and thylakoid membranes (Kumar et al., 2023).

Plant-beneficial bacteria have garnered significant interest for their potential applications in sustainable agriculture as biofertilizers or biopesticides as well as phytoremediation (Zulfiqar et al., 2021; Sheteiwy et al., 2022; Yang et al., 2022; El-Sawah et al., 2023; Yaghoubi Khanghahi et al., 2024). Notably, certain plant growth-promoting bacteria (PGPB) have been proposed as stress-tolerant bioactive strains. These bacteria not only enhance plant growth but also mitigate the adverse effects of environmental stressors by bolstering plant resistance and productivity (Kumar and Dubey, 2020; Madnay et al., 2022). PGPB can stimulate plant growth, increase yield, and prevent pathogen infections by enhancing the synthesis of phytohormones and antioxidants, which consequently impact physiological and metabolic characteristics (Mandon et al., 2021; Pankievicz et al., 2021). Among PGPB, *Micromonospora* sp., which have been isolated from diverse environmental locations, have attracted attention due to their capacity to produce phytohormones (Trujillo et al., 2014; Ortúzar et al., 2020). Moreover, *Micromonospora* sp. are recognized as significant sources of antibiotics (El-Tarabily et al., 2008), making them potent biocontrol agents against pathogenic fungi and stress responses (Zadel et al., 2020).

The production of natural bioactive metabolites, particularly those derived from microorganisms such as bacteria can help plants tolerate stress (Yaghoubi Khanghahi et al., 2022a; Athukorala et al., 2023). Environmental stressors are known to stimulate antioxidant production in living organisms, among which, heat stress is a primary abiotic challenge for both eukaryotes and prokaryotes (Huang et al., 1998; Larkindale and Huang, 2004), particularly in the context of global environmental changes. Heat stress induces the production of reactive oxygen species (ROS), leading to oxidative stress (Foyer et al., 1997; Kocsy et al., 2004). While the heat stress mechanisms in eukaryotes and prokaryotes like cyanobacteria are well-studied, the responses of bacteria to stress are less understood (Hassan et al., 2020). Moreover, to our knowledge, no studies have investigated the use of *Micromonospora* species to enhance wheat growth under stress conditions.

Therefore, the aim of the current research was to study the modifications in the biochemical, physiological, and antioxidant parameters of wheat plants under heat stress through an examination of the potential of plant growth-promoting bacteria. Efforts were also undertaken to enhance our understanding of how two distinct genotypes, one sensitive to heat and the other tolerant, respond to heat stress when exposed to beneficial bacterial inoculation. This research seeks to further our understanding of these interactions and develop strategies to protect plants from the adverse effects of future temperature increases.

## 2 Material and methods

### 2.1 Bacterial isolation and purification

Eighteen actinobacterial strains were isolated from Deseret soil located at Riyadh, Saudi Arabia. Soil (2 g) was serially diluted (1:10–1:1000) in sterile saline and plated on starch-casein agar (Küster and Williams, 1964) and soil extract media (Barakate et al., 2022) with glycerol (carbon source). Nalidixic acid was also added to inhibit bacteria capable of overcrowding, without inhibiting the growth of actinobacteria. Antifungals such as cycloheximide and nystatin (0.05 mg mL^-1^) were added to soil. Plates were incubated at 28°C for 7-12 days, and colonies were then purified and stored in 20% glycerol at −20°C as stock cultures.

### 2.2 Molecular identification and phylogenetic analysis

The selected potential actinobacterial isolate was subjected to extract the genomic DNA according to the technique outlined by Hong et al (Hong et al., 2009). The 16S rRNA gene PCR amplification was conducted on the template using the universal primers 27F (5’-AGAGTTTGATC(AC)TGCCTCAG-3’) and 1498R (5-ACGGCTACCTTGTTACGACTT-3). All PCR amplification was performed in reactions buffer contains total DNA, Taq DNA polymerase, 3 mM of dNTP, and 3 mM MgCl_2_. The PCR condition was set on an initial denaturation (94°C for 5 min) and 35-40 cycles of amplification (95°C for 1.2 min, 55°C for 1.2 min, and 72°C for 2.5 min), followed by an extension step (73°C for 4 min). The amplicons were sequenced from both ends by MacroGen Company (South Korea; http://www.dna.macrogen.com). The bacteria isolate was identified by aligning the sequences with the BLASTn tool in the NCBI database (www.ncbi.nlm.nih.gov). More details on Abdelgawad et al (AbdElgawad et al., 2023). The MEGAX software program was used to carry out the cluster analysis.

### 2.3 Determination of biological and phytochemical activities of the selected isolate

Antioxidant activity of the selected isolate was assessed with the ferric reducing antioxidant power (FRAP) assay, measuring absorbance at 590 nm, based on Schlesier et al (Schlesier et al., 2002). The total phenolic and flavonoid content in bacterial isolates was analyzed according to Holder and Boyce (1994). A 24-hour culture was used to inoculate the medium, followed by incubation (37°C for 7 days) in darkness. After incubation, samples were centrifuged at 6000 rpm for 10 minutes. This acidified supernatant (by HCl to pH 2.8) was mixed with an equal volume of diethyl ether and left to incubate in the dark for 4 hours. After overnight incubation at 4°C, the upper solvent phase was evaporated, and 2–3 mL of HPLC-grade methanol was added. Quantification of indole-3-acetic acid (IAA) and gibberellic acid (GA3) was conducted using high-performance liquid chromatography (HPLC). Cytokinin (CK) production by actinobacteria was assessed via a cucumber cotyledon greening bioassay, as outlined by Fletcher et al (Fletcher et al., 1982).

### 2.4 Wheat priming with bacterial suspension

Healthy uniform seeds of two genotypes of wheat (*Triticum aestivum*) including Giza-168 as a heat-tolerant genotype and Misr-3 as a heat-sensitive genotype (Omar et al., 2023) were planted in pots (with a diameter and height of 25 cm) containing sterilized clay soil (Tref EGO substrates, Netherland). The seeds were obtained from Agriculture Research Center, Giza, Egypt. The inoculation treatment is divided into two levels including (i) soil and seeds treatment with actinobacterial suspension and (ii) control treatment by adding sterile deionized water to the soil and priming the seeds with it. For biofertilization treatment, the bacterial strain was grown in Nutrient Broth medium at 28°C for 1 day and then was concentrated by centrifugation at 6500 rpm for 10 min, and the obtained pellet was washed and re-suspended in a sterile KCl solution (0.9%, w/v). The density of this bacterial suspension was adjusted to 10^–7^ CFU mL^−1^, approximating an optical density at 600 nm equivalent to 0.7–0.8, and used for seeds and soil inoculation before the initial planting and for subsequent additions to pots every 2 weeks (Yaghoubi Khanghahi et al., 2020). This suspension was applied to the soil before planting and added to the pot’s biweekly. Soil was autoclaved prior to bacterial inoculation treatment to eliminate unwanted microbial contaminants, thereby ensures that only targeted bacterial strains thrive during the incubation, minimizing interference from fast-growing or competing soil microorganisms.

Plants were also exposed to two levels of heat stress including controlled high-temperature conditions (38°C) and a recommended optimal temperature (24°C/18°C) for two weeks. The temperature applied was chosen after preliminary investigation, where initial tests using a wide range of temperatures and time period treatments reported 45°C to induce a progressively adverse impact with >50% dead plants after 14 days. The three pots represented three biological replicates for each treatment. All the pots were weighed daily to keep the water level (> 60% of field capacity). The shoots’ fresh weight (FW) and dry weight (DW) were gouged after seven weeks of growth and kept at -80°C for further examinations.

### 2.5 Determination of photosynthetic related parameters in wheat plants

Some photosynthetic traits were assessed to investigate the impacts of beneficial actinobacteria and heat stress on plant biomass production. In this regard, the light-saturated photosynthetic rate of the most youthful, expanded leaves was analyzes by a LI-COR portable photosynthesis system. The concentration of chlorophyll *a* (Chl *a*) and chlorophyll *b* (Chl *b*) pigments were measured by reading the absorbance of the extracted samples at 665 and 652 nm, respectively (Lichtenthaler and Buschmann, 2001). The activity of RuBisCO enzyme was assessed based on the oxidation of nicotinamide adenine dinucleotide at wave length of 340 nm (Lobo et al., 2015).

### 2.6 Determination of oxidative stress markers

To estimate malondialdehyde (MDA) concentration in the shoot tissues, the fresh samples were homogenized in ethanol (80% v/v) at 7000 g for in 60 second using a MagNALyser and reacted with thiobarbituric acid (TBA) reagent. The absorbance of the final product was measured at 450, 532, and 600 nm (Hodges et al., 1999). The content of hydrogen peroxide (H_2_O_2_) in the shoots was also measured in trichloroacetic acid (0.1% v/v) on the basis of peroxide-catalyzed oxidation of Fe^2+^, according to the xylenol orange method (AbdElgawad et al., 2016).

### 2.7 Sugars content

The sugars in the fresh shoot samples were extracted using a TAE buffer (50 mM, pH 7.5) supplemented with polyclar (0.15% v/v), sodium azide (0.02% v/v), PMSF (2 mM), sodium bisulfite (12 mM), mannitol (10 mM), and mercaptoethanol (1 mM). Following extraction, the samples were centrifuged, and the supernatant was passed through a mixed-bed Dowex column containing 300 µL each of Dowex H^+^ and Dowex Ac^–^ (100–200 mesh) (AbdElgawad et al., 2023). Quantification of glucose, sucrose, and fructose was conducted using high-performance anion-exchange chromatography with pulsed amperometric detection (HPAEC-PAD) according to the methodology described by AbdElgawad et al (AbdElgawad et al., 2023). Total soluble sugars in the shoot were quantified using a spectrophotometric method. Initially, sugars were extracted with 80% ethanol (v/v) and reacted with anthrone reagent (anthrone dissolved in 72% v/v H_2_SO_4_), following the protocol outlined by Yaghoubi et al (Yaghoubi Khanghahi et al., 2022a). The absorbance of the samples was measured at 625 nm using a multi-mode microplate reader (Synergy Mx, Biotek, Santa Clara, USA) to determine the total soluble sugar content.

### 2.8 Determination of the overall antioxidants

Total antioxidant capacity in the shoot samples was measured using ferric reducing/antioxidant power (FRAP) reagent, containing 0.3 M acetate buffer (pH 3.6), TPTZ (0.01) and FeCl_3_ (0.02 M), followed by reading the OD at 600 nm (Benzie and Strain, 1999). The standard was 6−hydroxy−2,5,7,8−tetramethylchromane−2−carboxylic acid (Trolox). The content of total polyphenols and flavonoids in the shoot samples, as described by Zhang et al (Zhang et al., 2006). and Chang et al (Chang et al., 2022), respectively. The total tocopherols by HPLC (SCL-10 AVP, Shimadzu Corporation) (AbdElgawad et al., 2015). HPLC method was employed to estimate reduced glutathione (GSH) and reduced ascorbate (ASC) (Hartley-Whitaker et al., 2001; Potters et al., 2004). The activities of antioxidant enzymes were assessed in a semi-high-throughput set-up (AbdElgawad et al., 2016; Shabbaj et al., 2022). The activity of superoxide dismutase (SOD) was measured by estimating the inhibition of nitro-blue tetrazolium reduction (Dhindsa et al., 1982). The oxidation of pyrogallol in phosphate buffer at 430 nm was analyzed to measure the activity of peroxidase (POX) (Kumar and Khan, 1983). The activity of catalase (CAT) was assessed by observing the H_2_O_2_ decomposition at 240 nm (Kumar and Khan, 1983). The estimation of ascorbate peroxidase (APX) activity was done by recording the decrease in absorbance at 290 nm as fully described by Nakano and Asada (1981). The decrease in NADPH at 340 nm was monitored to estimate the activity of glutathione reductase (GR) (Murshed et al., 2008). The reduction of 2-hydroxy-ethyl-disulfide by reduced glutathione was recorded to measure the activity of glutaredoxin (Grx) (Lundberg et al., 2001). The reduction in NADPH absorption was evaluated to measure the activity of glutathione peroxidase (GPX) (Drotar et al., 1985). Thioredoxin (Trx) content was assayed by recording NADPH oxidation at 340 nm (Wolosiuk et al., 1979).

### 2.9 Determination of anthocyanins, phenolics and flavonoids

To measure the anthocyanins content, methanol: HCl (99:1 v/v) was used for homogenizing the samples, followed by incubating the extracted samples at 25°C for 24 h in the dark conditions and centrifuging at 4000 g for 5 min. The absorbance of the extracted supernatant was read at 550 nm (ε550 = 233,000 M^−1^ cm^−1^) (Wagner, 1979).

Flavonoid compounds and phenolic acids were analyzed by HPLC with a Lichrosorb Si-60 column (7 μm, 3 × 150 mm) and DAD detector, as fully explained by Hamad et al (Hamad et al., 2015). Briefly, acetone:water (4:1, v/v) solution was used for homogenizing the shoot samples for 24 h. the final extraced samples were subjected to HPLC at 0.8 mL min^-1^ (flow rate), in which the mobile phase consisted of water:formic acid (90:10 v/v) and acetonitrile:water:formic acid (85:10:5 v/v/v). A calibration curve of the related standard was considered to estimate the content of each compound.

The activity of phenylalanine amino-layse (PAL) was measured by extracting the samples in sodium borate buffer and reading the absorbance of trans-cinnamic acid output at 290 nm (Hamad et al., 2015). Cinnamate (CA) 4-hydroxylase (C4H) activity was measured based on a method described by Jadhav et al (Jadhav et al., 2013). Briefly, shoot samples were homogenized in Tris buffer and its absorbance was recorded at 340 nm (ε340 = 6200 M^−1^ cm^−1^). 4-coumarate:coenzymeA ligase (4CL) activity was measured spectrophotometrically to measure formation of CoA esters of various phenolic acids (Allina et al., 1998).

### 2.10 Statistical analysis

Statistical analyses were performed using the SPSS statistical package (SPSS Inc., Chicago, IL, USA). One-way analysis of variance (ANOVA) and Tukey’s Honest Significant Difference (HSD) *post-hoc* test were conducted to determine significance at a 5% probability level (*p*<0.05). The values represent the mean of at five replicates.

## 3 Results

### 3.1 Bacterial identification

**Figure 1:**
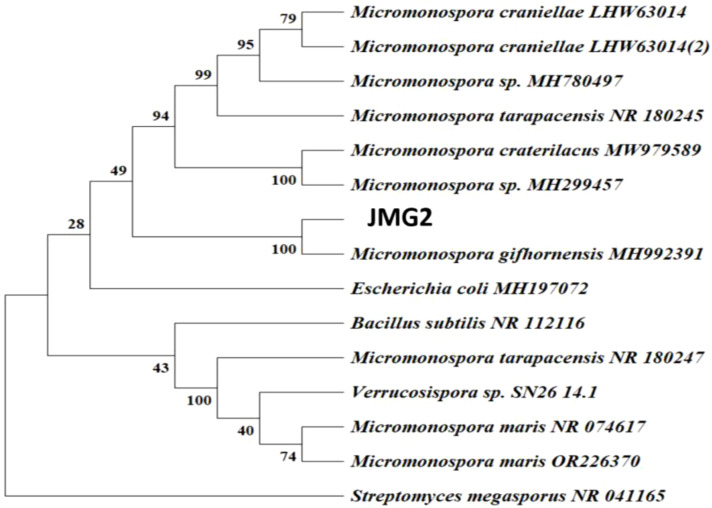
Phylogenetic analysis of JMG2. The tree was constructed by the neighbor-joining method based on 16S rRNA sequences. Numbers refer to bootstrap values for each node out of a total of 100 replicate resampling. The numbers in the bracket are the EMBL accession numbers of the 16S rRNA sequences of reference bacteria. *Streptomyces megaspores*, *Bacillus subtilis* and *Escherichia coli* were used as the outgroup.

Among several bacterial isolates from Riyadh, Saudi Arabia, one isolate that showed a great growing and colonizing ability under heat stress conditions (56°C) in the primary experiments was selected for further analysis. Based on the 16S rRNA gene sequencing, the actinobacteria isolate was closely related to *Micromonospora* sp, with similarity ≥ 100% (**Figure 1**). The NCBI and GenBank nucleotide sequence databases received the 16S rRNA gene data under the accession number (OQ978135) for deposit.

### 3.2 Biological activity and phytochemical and *Micromonospora* sp. strain

*Micromonospora* sp. showed high antioxidant bioactivity as indicated by FRAP assay (**Table 1**). The strain can also produce high amounts of the total phenolic and flavonoid metabolites. This bioactive strain also showed a high potential for phytohormone production such as indole acetic acid (IAA), gibberellic acid (GA), and cytokinin (CK) (**Table 1**).

**Table 1:** Antioxidant activity and antioxidant and phytohormones production of *Micromonospora* sp.

Measurements	Concentrations
Antioxidant activity (FRAP) (μmole Trolox g^−g^ DW)	78.0 ± 5.2
Total phenols (mg gallic acid g^−1^ DW)	15.7 ± 1.7
Total flavonoids (mg quercetin g^−1^ DW)	8.9 ± 0.87
IAA (mg g^−1^ DW)	6.1 ± 0.35
GA (mg g^−1^ DW)	2.6 ± 0.72
CK (mg g^−1^ DW)	0.57 ± 0.1

### 3.3 Plant biomass and photosynthetic parameters

**Figure 2:**
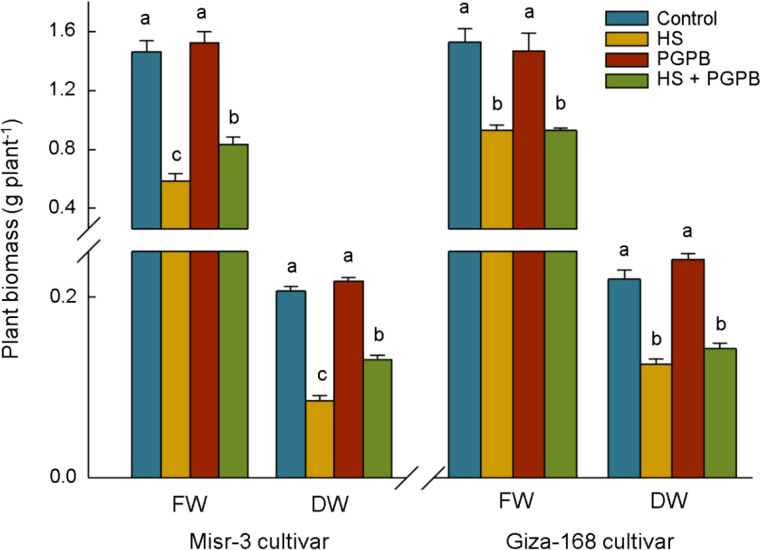
The effect of *Micromonospora* sp. and heat stress (HS) on plant biomass in two heat-sensitive (Misr-3) and tolerant (Giza-168) cultivars. The means in each parameter and each cultivar with similar small letter(s) are not significantly different at 5% probability level (Tukey HSD test). FW, Fresh weight; DW, Dry weight.

**Figure 3:**
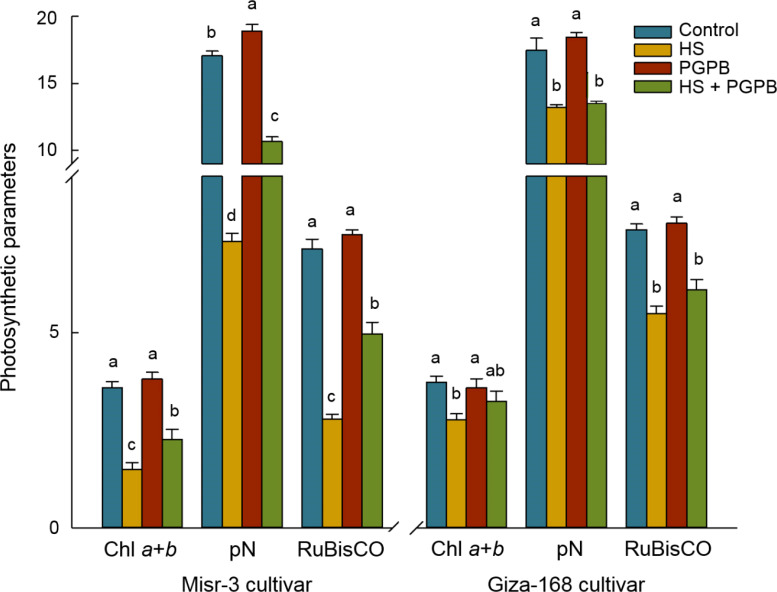
The effect of *Micromonospora* sp. and heat stress (HS) on the photosynthesis parameters (µmol CO_2_ m^-2^ s^-1^ for P_N_, µg g^-1^ FW for chlrophyll *a*+*b* content, and mg g^-1^ protein for RuBisCO activity) in two heat-sensitive (Misr-3) and tolerant (Giza-168) cultivars. The means in each parameter and each cultivar with similar small letter(s) are not significantly different at 5% probability level (Tukey HSD test). P_N_, Photosynthesis rate.

Plants exposed to stress had the lower biomass weight in both cultivars (*p* < 0.05), a clear improvement in fresh (+43%) and dry weights (+53%) was observed in sensitive cultivar when they were treated with *Micromonospora* sp. under heat stress conditions, the improvement that was not observed in the stress-tolerant line (*p* > 0.05) (**Figure 2**). Heat stress conditions led to a sharp decrease (*p* < 0.05) in the photosynthesis rate, Chl *a*+*b* content, and RuBisCO activity, equal to -57%, -58%, and -61% in the sensitive cultivar, and -26%, -24%, and -28% in the tolerant cultivar compared the control un-stressed plants, respectively (**Figure 3**). Nevertheless, *Micromonospora* sp. treatment improved (*p* < 0.05) the status of the mentioned parameters under stress in the sensitive line by +51%, +48%, and +78% compared to the non-inoculated plants, respectively. This increase in the inoculated tolerant cultivar was not significant (*p* > 0.05).

### 3.4 Oxidative stress markers

**Figure 4:**
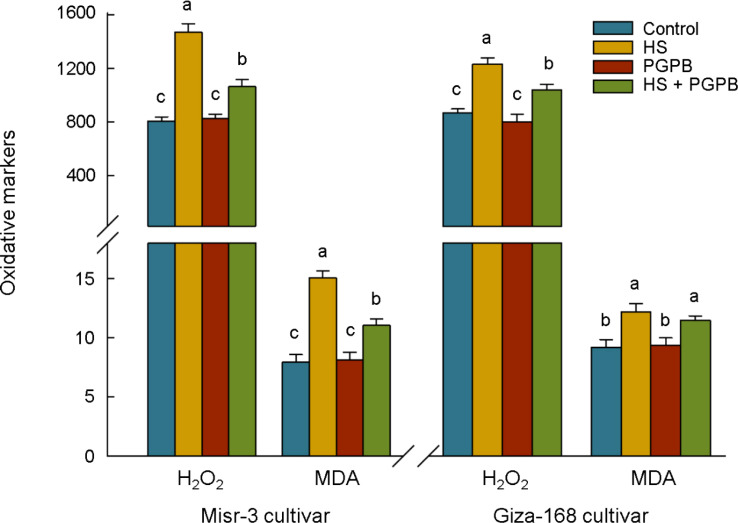
The effect of *Micromonospora* sp. and heat stress (HS) on the oxidative markers (represented as µmol g^-1^ FW for H_2_O_2_ and nmol g^-1^ FW for MDA) in two heat-sensitive (Misr-3) and tolerant (Giza-168) cultivars. The means in each parameter and each cultivar with similar small letter(s) are not significantly different at 5% probability level (Tukey HSD test). H_2_O_2_, Hydrogen peroxide; MDA, Malondialdehyde.

Oxidative stress was increased in both cultivars grown under heat stress conditions (*p* < 0.05), where the concentration of H_2_O_2_ and MDA were 82% and 90% in sensitive cultivar and 42% and 32% in tolerant cultivar higher than those un-stressed plants, respectively (**Figure 4**). The most interesting aspect of the results is the decreasing trend (*p* < 0.05) in H_2_O_2_ and MDA contents in response to bio-inoculation treatment in stressed plants about -28% and -27% in sensitive cultivar, respectively. This reduction was 16% (*p* < 0.05) and 6% (*p* > 0.05) in the stress-tolerant cultivar, respectively.

### 3.5 Antioxidant system

**Figure 5:**
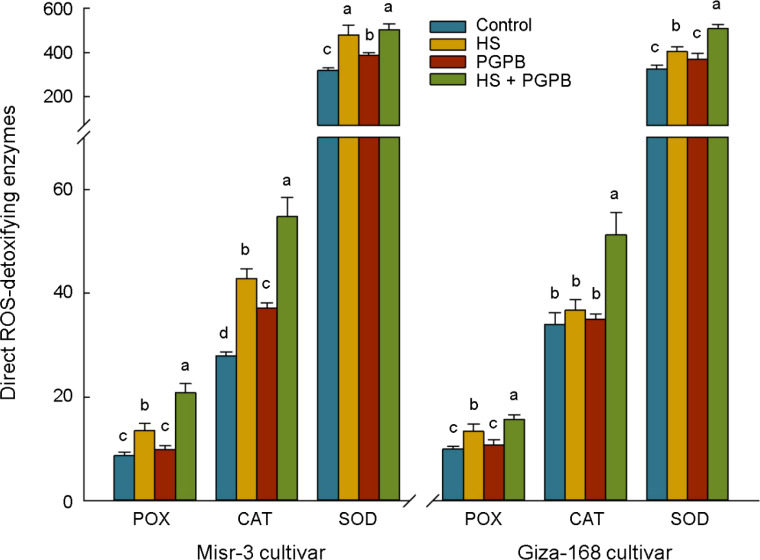
The effect of *Micromonospora* sp. and heat stress (HS) on the antioxidant direct ROS-scavenging enzymes (represented as μmol min^-1^ mg^-1^ protein for POX and CAT, and mmol min^-1^ mg^-1^ protein for SOD) in two heat-sensitive (Misr-3) and tolerant (Giza-168) cultivars. The means in each parameter and each cultivar with similar small letter(s) are not significantly different at 5% probability level (Tukey HSD test). POX, Peroxidase; CAT, Catalase; SOD, Superoxide dismutases.

**Figure 6:**
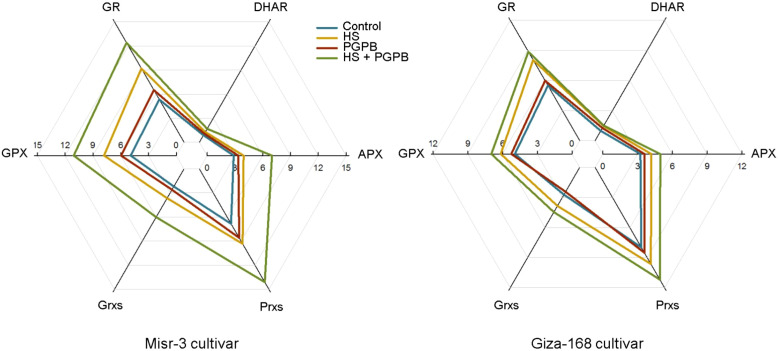
The effect of *Micromonospora* sp. and heat stress (HS) on the enzymatic components (μmol min^-1^ mg^-1^ protein) of the ascorbate-glutathione (ASC/GSH) and catalytic cycles in two heat-sensitive (Misr-3) and tolerant (Giza-168) cultivars. GPX, Glutathione peroxidase; APX, Ascorbate peroxidase; GR, Glutathione reductase; Grxs, Glutaredoxins; Prxs, Peroxiredoxins; DHAR, Dehydroascorbate reductase.

The activity of the direct ROS-detoxifying enzymes (POX, SOD, and CAT) and those enzymatic components of the ascorbate-glutathione (ASC-GSH) pathway (APX, GPX GR, and DHAR), as well as other antioxidant enzymes involved in the catalytic cycle (Prxs and Grxs) were investigated to unveil the plant mechanisms in promoting the toleration against oxidative damage induced by heat stress, and relieving the content of H_2_O_2_ and MDA in plants (**Figures 5**, **6**). Accordingly, however, the activity of SOD, POX and CAT were significantly increased (except for CAT in tolerant cultivar) under stress conditions, they were more activated (*p* < 0.05) in *Micromonospora* sp.-treated plants in both cultivars (except for SOD in tolerant cultivar) compared to the no inoculation treatment in stressed plants (**Figure 5**). Similarly, the highest activity of APX, DHAR, GR, GPX, Grxs, and Prxs in both cultivars was observed in stressed plants treated with *Micromonospora* sp., which in the sensitive plants were 78, 30, 35, 42, 65, and 51% greater than those in non-inoculated stressed plants (*p* < 0.05). In contrast, this increase was more imperceptible in the tolerant cultivar, so only the activity of APX (+19%), GPX (+13%), and Prxs (+16%) was significantly higher than those non-inoculated plants under stress, and the increase in the activity of DHAR (+3%), GR (+10%), and Grxs (+16%) was not statistically significant (*p* > 0.05) (**Figure 6**).

**Figure 7:**
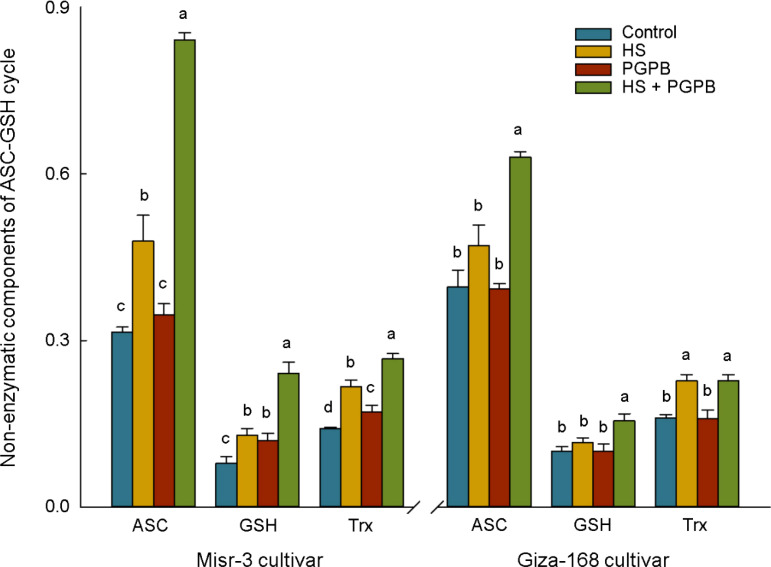
The effect of *Micromonospora* sp. and heat stress (HS) on non-enzymatic (µmol g^-1^ FW) component of the ascorbate-glutathione (ASC/GSH) cycle and redox-dependent signaling pathway in two heat-sensitive (Misr-3) and tolerant (Giza-168) cultivars. The means in each parameter and each cultivar with similar small letter(s) are not significantly different at 5% probability level (Tukey HSD test). ASC, Ascorbate; GSH, Gluthatione; Trx, Thioredoxin.

**Figure 8:**
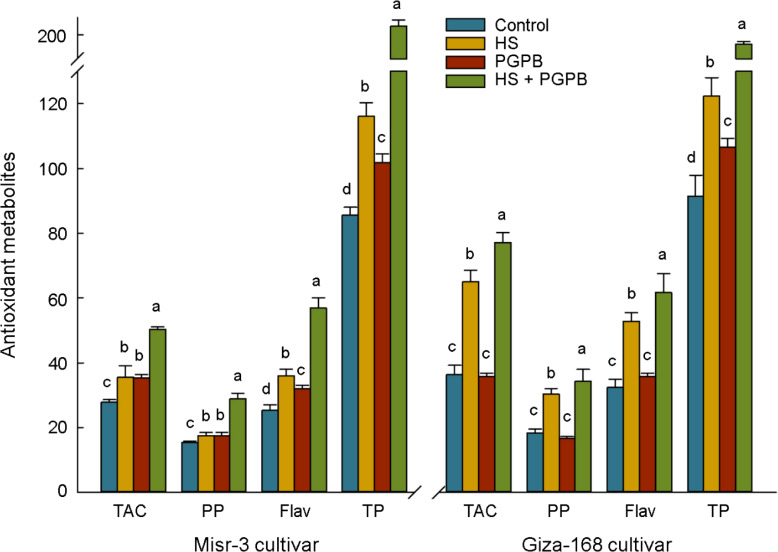
The effect of *Micromonospora* sp. and heat stress (HS) on the antioxidant metabolites in plant (µmol Torolex g^-1^ FW for TAC; mg GAE g^-1^ FW for PP; mg Quercetin g^-1^ FW for Flav; ng g^-1^ FW for TP) in two heat-sensitive (Misr-3) and tolerant (Giza-168) cultivars. The means in each parameter and each cultivar with similar small letter(s) are not significantly different at 5% probability level (Tukey HSD test). TAC, Total antioxidant capacity; PP, Polyphenols; Flav, Flavonoids; TP, Tocopherols.

The content of antioxidant proteins in the ASC/GSH pathway (GSH and ASC) and redox-dependent signaling pathway (Trxs) in the sensitive cultivar were significantly increased in response to both bio-inoculation and stress treatments (*p* < 0.05) (**Figure 7**). Accordingly, the highest content of ASC, GSH, and Trxs in inoculated plants under stress was 76, 86, and 23% (*p* < 0.05) greater than those in non-inoculated stressed plants. On the other hand, the content of ASC and GSH in the tolerant cultivar was not affected by stress (*p* > 0.05), however, the values in *Micromonospora* sp.-treated plants under stress reached the highest and were about +34-61% higher than other treatments (*p* < 0.05). Similarly, *Micromonospora* sp. further improved the total antioxidant capacity (TAC) and antioxidants production (PP, Flav, TP), particularly under heat stress (**Figure 8**).

### 3.6 Carbohydrates metabolism

**Figure 9:**
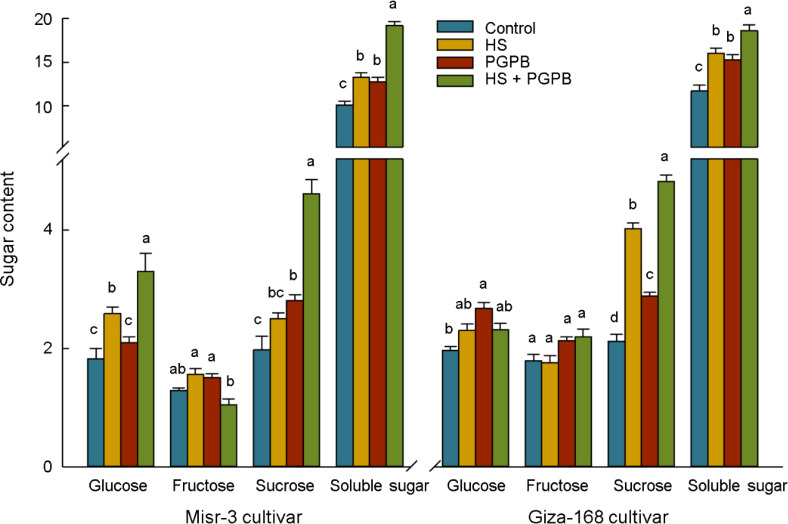
The effect of *Micromonospora* sp. and heat stress (HS) on sugar content (mg g^-1^ FW) in two heat-sensitive (Misr-3) and tolerant (Giza-168) cultivars. The means in each parameter and each cultivar with similar small letter(s) are not significantly different at 5% probability level (Tukey HSD test).

To investigate carbon metabolism in wheat plants, the adaptation and modification in soluble sugars content were studied under heat stress and bio-inoculation treatment (**Figure 9**). In this regard, heat stress remarkably (*p* < 0.05) enhanced the content of total soluble sugar by 31% and 37% in the heat-sensitive and –tolerant cultivars compared to the control treatment, respectively. Moreover, *Micromonospora* sp. treatments greatly (*p* < 0.05) improved total soluble sugar content in both un-stressed and stressed plants, which were 26% and 45% in sensitive cultivar and 31% and 16% in tolerant cultivar higher than those non-inoculated plants under same stress level, respectively (**Figure 9**). Interestingly, by investigating the three main forms of soluble sugars in plants, a diverse reaction of fructose, glucose, and sucrose was found (**Figure 9**). Accordingly, fructose content did not show a specific response to stress and bio-inoculation in both cultivars. In contrast, the content of glucose in the sensitive cultivar was significantly (*p* < 0.05) increased in response to bio-inoculation in both stressed (+28%) and un-stressed (+15%) plants, while increasing only in un-stressed (+36%) tolerant cultivar (*p* < 0.05). The highest accumulation of sucrose was observed in bio-inoculated plants under stress in both cultivars, which were significantly higher than non-inoculated plants under stress and non-stress conditions, equal to +85% and +134% in sensitive cultivar and +20% and +127% in tolerant cultivar, respectively (**Figure 9**).

### 3.7 Anthocyanin metabolism

The modifications in the content of anthocyanin and the intermediates in their production, as well as some enzymes involved in their synthesis pathway in response to *Micromonospora* sp. and/or heat stress treatments were evaluated (**Table 2**). Interestingly, there were differences in the responses of anthocyanins to the treatments in each cultivar. Accordingly, anthocyanin content was increased by *Micromonospora* sp. in stressed sensitive cultivar (+28%; *p* < 0.05), while remained unchanged in the tolerant cultivar (+5%; *p* > 0.05) compared to no inoculation treatment under stress (**Table 2**). The content of individual phenolic acids, cinnamic acid and coumaric acid, in sensitive cultivar decreased in response to the inoculation treatment under both stress and control treatments, while only coumaric acid showed a similar reaction in the tolerant cultivar (*p* < 0.05) and no significant difference was observed in the content of cinnamic acid (*p* > 0.05).

**Table 2:** The effect of *Micromonospora* sp. and heat stress (HS) on some phenolic acids (mg g^-1^ DW), flavonoid compounds (mg g^-1^ DW), phenylalanine amino acid (mg g^-1^ protein), phenylalanine amnio lyase (PAL) enzyme activity (nkatal mg^-1^ protein) and enzymes involved in anthocyanin metabolism (nmol mg^-1^ protein min^-1^) in two heat-sensitive (Misr-3) and tolerant (Giza-168) cultivars.

	Misr-3 cultivar				Giza-168 cultivar			
	Control	HS	PGPB	HS + PGPB	Control	HS	PGPB	HS + PGPB
Anthocyanin	3.70 ± 0.43 b	3.91 ± 0.49 b	4.52 ± 0.09 ab	5.02 ± 0.13 a	4.09 ± 0.45 b	5.90 ± 0.56 a	3.84 ± 0.45 b	6.21 ± 0.45 a
Phenylalanine	1.16 ± 0.09 b	1.17 ± 0.09 b	1.57 ± 0.17 a	1.59 ± 0.13 a	1.60 ± 0.25 b	1.58 ± 0.24 b	1.75 ± 0.26 b	2.42 ± 0.24 a
Cinnamic acid	8.70 ± 0.17 a	8.20 ± 0.16 ab	7.42 ± 0.14 b	7.37 ± 0.14 b	10.08 ± 0.19 a	10.01 ± 0.19 a	9.70 ± 0.17 a	10.51 ± 0.17 a
Coumaric acid	2.77 ± 0.08 a	3.15 ± 0.10 a	1.89 ± 0.03 b	1.73 ± 0.04 b	5.18 ± 0.10 a	4.84 ± 0.10 a	3.93 ± 0.06 b	3.14 ± 0.26 b
Naringenin	1.69 ± 0.05 a	1.29 ± 0.22 a	1.60 ± 0.24 a	1.44 ± 0.21 a	1.67 ± 0.15 a	1.96 ± 0.12 a	1.84 ± 0.14 a	1.98 ± 0.14 a
PAL	10.78 ± 1.15 b	10.79 ± 0.87 b	12.99 ± 0.99 a	12.30 ± 1.15 a	20.77 ± 2.11 b	23.94 ± 1.88 a	19.40 ± 1.38 b	24.62 ± 1.38 a
CHS	3.59 ± 0.19 b	3.35 ± 0.20 b	5.13 ± 0.33 a	4.71 ± 0.37 a	6.84 ± 0.70 b	8.30 ± 0.63 a	6.90 ± 0.45 b	8.11 ± 0.45 a
C4H	0.54 ± 0.05 a	0.47 ± 0.05 c	1.00 ± 0.02 a	0.70 ± 0.02 b	0.86 ± 0.04 b	1.54 ± 0.17 a	0.70 ± 0.06 b	1.74 ± 0.13 a
4CL	0.85 ± 0.02 a	0.68 ± 0.02 a	0.87 ± 0.02 a	0.79 ± 0.02 a	1.52 ± 0.15 a	1.27 ± 0.23 b	1.49 ± 0.15 a	1.36 ± 0.17 ab

The means in each parameter and each cultivar with similar small letter(s) are not significantly different at 5% probability level (Tukey’s HSD test). PAL, Phenylalanine ammonia lyase; CHS, Chalcone synthase; C4H, cinnamate (CA) 4-hydroxylase; 4CL, 4-coumarate:coenzymeA ligase.

No differences were found in the content of naringenin and the activity 4CL in both cultivars (*p* > 0.05) (**Table 2**). Nevertheless, heat stress significantly improved PAL (+15-27%), CHS (+18-21%), and C4H (+57-149%) activities in the tolerant cultivar in both *Micromonospora* sp. and no inoculation treatments. In contrast, *Micromonospora* sp. improved PAL and CHS activity in the sensitive cultivar under both stress (+14% and +28%, respectively) and non-stress conditions (+21% and +43%, respectively). Moreover, in addition to the stress, *Micromonospora* sp. inoculation also improved the C4H activity (+49-85%) in the sensitive cultivar under stress. The highest content of phenylalanine in both cultivars belonged to the bio-inoculation treatment under stress, which was significantly higher than that in no inoculation sensitive (+36%) and tolerant (+53%) cultivars under stress (**Table 2**).

## 4 Discussion

The current research tried to clear some of the unanswered questions concerning the consequences of global warming on plants, one of which is the rising air temperature level. Accordingly, the responses of two different heat-sensitive and -tolerant cultivars of wheat to heat stress were investigated, especially to show whether the application of a heat-tolerant *Micromonospora* sp. strain could relieve the adverse effects of stress through the modifications of some of the plant’s biochemical and physiological traits. The results clearly showed that the plant biomass significantly decreased under stress in both cultivars. Moreover, the biomass accumulation in sensitive cultivar significantly improved when expose to *Micromonospora* sp. inoculation treatment under stress, while the improvement was not observed in the stress-tolerant line. To explain the utilized strategies by plants, we foremost debated the response of photosynthetic traits.

Decline in photo-assimilates synthesizing in the source tissue under heat stress and consequently their lower translocation and accumulation rates in the sink tissue have been considered as the main reason of decreasing in biomass (Kumar et al., 2023). The decrease in the photosynthesis rate under heat stress in both cultivars in the present research has been previously ascribed to the less activity of RuBisCO and consequently, disruption of the chloroplast structure and function (Ashraf, 2022). It has been reported that the rising chlorophyll concentration in stressed plants can protect the function and structure of the chlorophyll system from degradation and disruption (Albqmi et al., 2023). Thus, it can be pointed out that the higher chlorophyll *a*+*b* content in the *Micromonospora* sp.-inoculated plants under stress can be a reason for the higher photosynthesis rate and therefore biomass accumulation in heat-sensitive cultivar compared to that tolerant. Likewise, it has already been reported that more activation of RuBisCO in stressed plants is one of the functional and non-stomatal traits in the Calvin cycle (Acosta-Motos et al., 2017) which in turn can be one of the main causes of improved photosynthetic rate in the sensitive cultivar under stress when treated with *Micromonospora* sp. Similar findings have already proved that the *Micromonospora* sp. treatment can boost plant tolerant against stress systematically (Meena et al., 2019) by employing some strategies such as the shift in soil-plant system and affecting supplying/uptaking soil nutrients (Yaghoubi Khanghahi et al., 2018), synthesizing of phytohormones (Yaghoubi Khanghahi et al., 2022b), and modulating the expression of some specific genes (Hagaggi and Mohamed, 2020).

Shift in carbohydrate metabolism was another strategy of stressed plants, particularly when they were treated with *Micromonospora* sp. An increment in soluble sugar content in heat-stressed plants for osmotic adjustment has been already reported (Harsh et al., 2016). No significant changes in the content of fructose and glucose in the tolerant cultivar may be due to the milder heat stress for this cultivar compared to the sensitive cultivar, as previously reported by Sassi-Aydi et al (Sassi-Aydi et al., 2014). Likewise, differences in the synthesis of individual sugars compared to others under stress have previously been documented (Thompson et al., 2017). In this regard, different responses of hexose sugars (e.g., glucose) and sucrose to environmental stress were attributed to various source and sink activities in plants during the developing stages (Rogers et al., 2004). Glucose, sucrose, and fructose are produced in source organs as the main soluble sugars, among which sucrose is then forwarded to the sink organs (Ma et al., 2021). Nevertheless, the improvement in photosynthesis rate in plants treated with *Micromonospora* sp. under stress compared to those non-inoculated, as shown in **Figure 2**, maybe the primary reason for an increase in carbohydrate synthesis and accordingly for adaptations in carbon metabolism (Thompson et al., 2017). Similarly, higher photosynthesis rate and activation of RuBisCO are considered the main factors improving carboxylation rate under stress (Drake et al., 1997), as was observed in the present study in the *Micromonospora* sp.-inoculated plants (sensitive cultivar) in stress conditions.

More concentrations of oxidative damage markers, including MDA in heat-stressed plants can represent a high level of ROS and consequently oxidative stress (Liu et al., 2020). This finding confirms the reports of previous research, in which oxidative stress was observed in heat-stressed plants, principally since the high level of such oxidative markers can surpass the valence of the antioxidant enzymatic reservoir (Hassan et al., 2020; Fortunato et al., 2023). Moreover, an obvious reduction tendency in the production of these oxidative damage markers was observed in *Micromonospora* sp. inoculation under stress, particularly in the sensitive cultivar (**Figure 4**). This result confirms the results of a great deal of the earlier studies, in which the favorable consequences of *Micromonospora* sp. inoculation in diminishing the accumulation of oxidative markers in heat-stressed plants have been reported (Khan et al., 2020b; Khan et al., 2020a). It seems possible that these results are due to the higher detoxification of ROS in plants treated with *Micromonospora* sp. under stress, in which the antioxidant molecules and enzymes were more activated compared to non-inoculated stressed plants.

It has been proved that the detoxification of overproduced ROS in plants is attributed to the activation of the antioxidant defense systems (Zarbakhsh et al., 2020; Shabbaj et al., 2022), particularly in *Micromonospora* sp.-treated plants (Madnay et al., 2022). Similar reports indicated a greater activity of direct ROS-detoxifying enzymes (CAT, SOD and POX) and those metabolites and enzymes in the ASC/GSH and catalytic pathways (GSH, ASC, Trxs, APX, GR, DHAR, GPX, Grxs, and Prxs) can alleviate the adverse effects of heat stress (Albqmi et al., 2023; Zarbakhsh et al., 2024). In this regard, the response of non-enzymatic component of the ASC-GSH pathway (ASC and GSH) in *Micromonospora* sp. treatment in the current research, agreed with the activation of involved enzymes, mainly APX, which catalyze the reduction of H_2_O_2_ into H_2_O (Madnay et al., 2022; Zarbakhsh et al., 2024). Similarly, activating the ASC-GSH pathway in PGPB treatment was assumed a primary approach for detoxifying ROS and preserving plant cells from oxidative damage (Carreiras et al., 2023; Zarbakhsh et al., 2024).

Our results also indicated the highest levels of total antioxidant capacity and antioxidant molecules (total flavonoids, polyphenols and total tocopherols) in heat-stressed plants, particularly in *Micromonospora* sp. treatment. These findings agree with the findings of other research, in which a high content of antioxidant metabolites in *Micromonospora* sp.-inoculated plants under different environmental stress has been reported as an adaptation strategy of plants under stress conditions (Yaghoubi Khanghahi et al., 2022a; Sarraf et al., 2023; Hagagy and AbdElgawad, 2024), in particular via the preserving the photosynthetic apparatus (Albqmi et al., 2023). The observed increase in these antioxidant metabolites content in *Micromonospora* sp. -treated plants could be attributed to the higher content of phenylalanine. This amino acid has an essential role as a precursor of many molecules in defense systems against environmental stress such as flavonoids, polyphenols, and anthocyanins (Pascual et al., 2016; Shomali et al., 2022). Moreover, the responses of phenylalanine and anthocyanins in this study corroborate the shifts in the activity of the phenylalanine ammonia-lyase (PAL) enzyme, which is responsible for catalyzing the deamination of phenylalanine as the foremost and rate-limiting stage of the phenylpropanoid pathway at the regulation point between primary and secondary metabolites (Fan et al., 2022). The similar reaction of cinnamate (CA) 4-hydroxylase (C4H), the enzyme catalyzing the second step of the phenylpropanoid pathway (Schoch et al., 2002), to the *Micromonospora* sp. treatment in the sensitive cultivar, and the heat stress in the tolerant cultivar (**Table 2**) confirms the activation of this pathway in the synthesizing of defense molecules. Similarly, it is clear that *Micromonospora* sp. could improve chalcone synthase (CHS) activity in the sensitive cultivar, which is known for its function as a major hub for the employed enzymes in the flavonoid synthesis pathway (Crosby et al., 2011). On the other hand, it seems possible that the heat stress alone in the tolerant cultivar was enough stimulus to increase the activity of PAL and CHS enzymes, the stimulating effect that was not observed in the sensitive cultivar. The lack of significant changes in naringenin content, as a product of the flavonoid pathway, in both cultivars can indicate the lack of non-competitively inhibition by this pathway in increasing the enzyme activity (Hinderer and Seitz, 1985), since it is believed that high accumulation of naringenin in the cytosol can limit CHS activity to avoid toxic levels in the cells (Whitehead and Dixon, 1983). The findings represented the regulative role of these detoxifying metabolites in the present study, particularly in *Micromonospora* sp. treatment, as one of the leading defense mechanisms under heat stress.

## 5 Conclusion

In conclusion, this study demonstrates that plant growth-promoting bacteria (*Micromonospora* sp.), play a significant role in mitigating the adverse effects of heat stress on wheat plants by enhancing photosynthetic efficiency, antioxidant defense, and metabolic adaptations. The sensitive wheat cultivar, under heat stress, exhibited substantial improvements in photosynthesis rate, chlorophyll content, RuBisCO activity, and biomass production when treated with *Micromonospora* sp., compared to untreated stressed plants. Additionally, *Micromonospora* sp. treatment notably reduced oxidative stress markers and enhanced the activity of antioxidant enzymes and the ASC-GSH pathway enzymes. The increased levels of antioxidant proteins, metabolites, and the activation of the phenylpropanoid pathway further contributed to reducing oxidative damage. These findings underscore the potential of utilizing *Micromonospora* sp. to develop heat-resilient wheat cultivars, offering a promising strategy for sustainable agriculture in the face of rising global temperatures. However, the study has limitation such as the experiment was conducted in a lab setting using sterile soil, which does not fully replicate the complex microbial interactions present in natural soil environments. The use of sterilized soil may oversimplify the interactions between *Micromonospora* sp. and wheat plants, as native soil microorganisms could significantly impact the efficacy of *Micromonospora* sp. treatments in field conditions. Future studies should also consider employing advanced genomic and transcriptomic approaches to elucidate the pathways influenced by bacterial inoculation. Finally, long-term effects of bacterial treatments on wheat resilience under varying environmental conditions have not been fully explored, highlighting the importance of conducting extended field trials to assess the durability of these treatments.

## Data Availability

The data presented in this study have been deposited in GenBank (NCBI) with the following accession number: PQ670967.
