# Supplementary material for: Inoculation with Micromonospora sp. enhances carbohydrate and amino acid production, strengthening antioxidant metabolism to mitigate heat stress in wheat cultivars
Source: Front Plant Sci. 2024 Dec 19;15:1500894. doi: 10.3389/fpls.2024.1500894 (PMC11696539; doi:10.3389/fpls.2024.1500894)
Supplement: Supplementary file 1 [file Table1.docx]

*
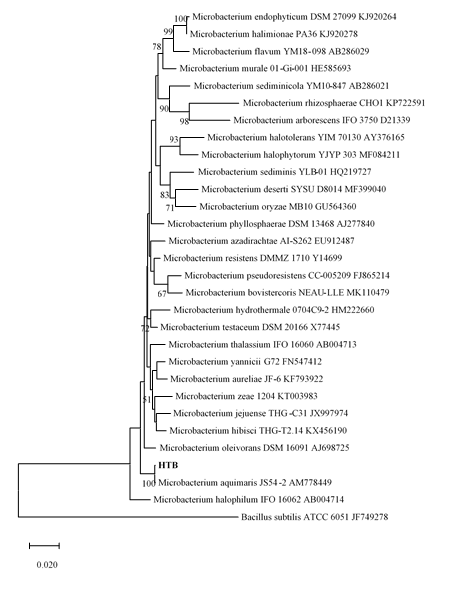
*

**Supplementary Fig. S1.** Neighbor-joining phylogenetic tree using MEGA-X program based on the 16S rRNA gene sequences of the isolate HTB and representatives of related taxa.
